# Supplementary material for: ITS Metabarcoding Reveals the Effects of Oregano Essential Oil on Fusarium oxysporum and Other Fungal Species in Soil Samples
Source: Plants (Basel). 2022 Dec 22;12(1):62. doi: 10.3390/plants12010062 (PMC9824880; doi:10.3390/plants12010062)
Supplement: Supplementary file 1 [file plants-12-00062-s001.zip › plants-2087223-supplementary.pdf]

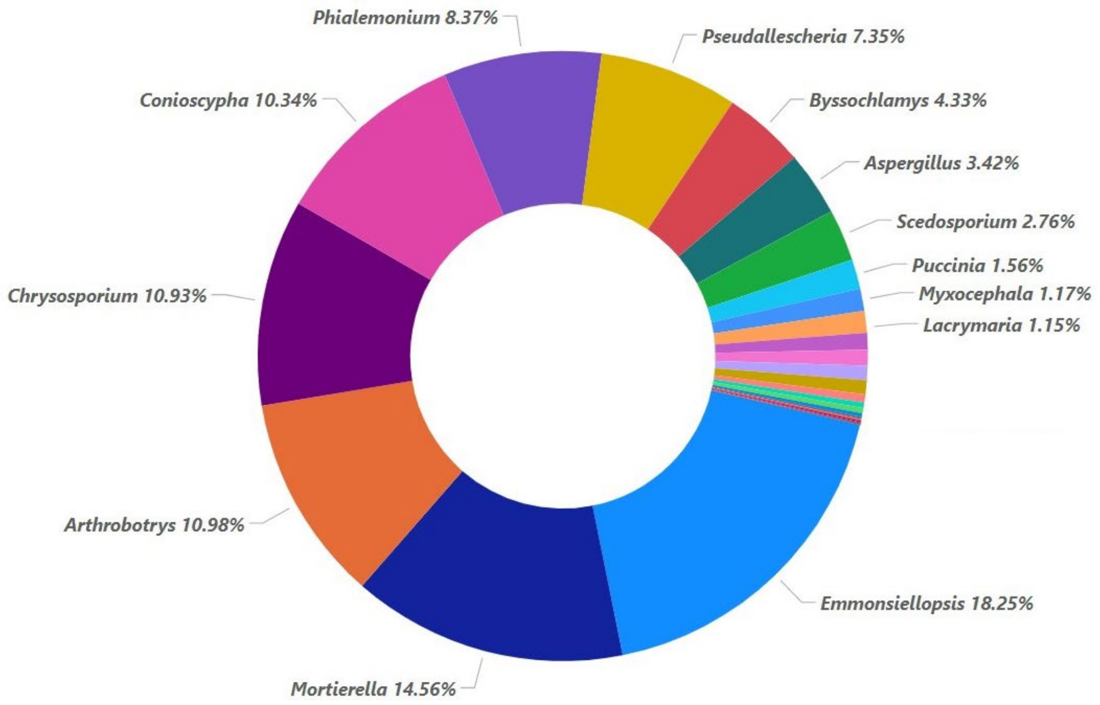

Figure S1. Relative abundance with percentages above 1% for Genera taxa, for sample A0.

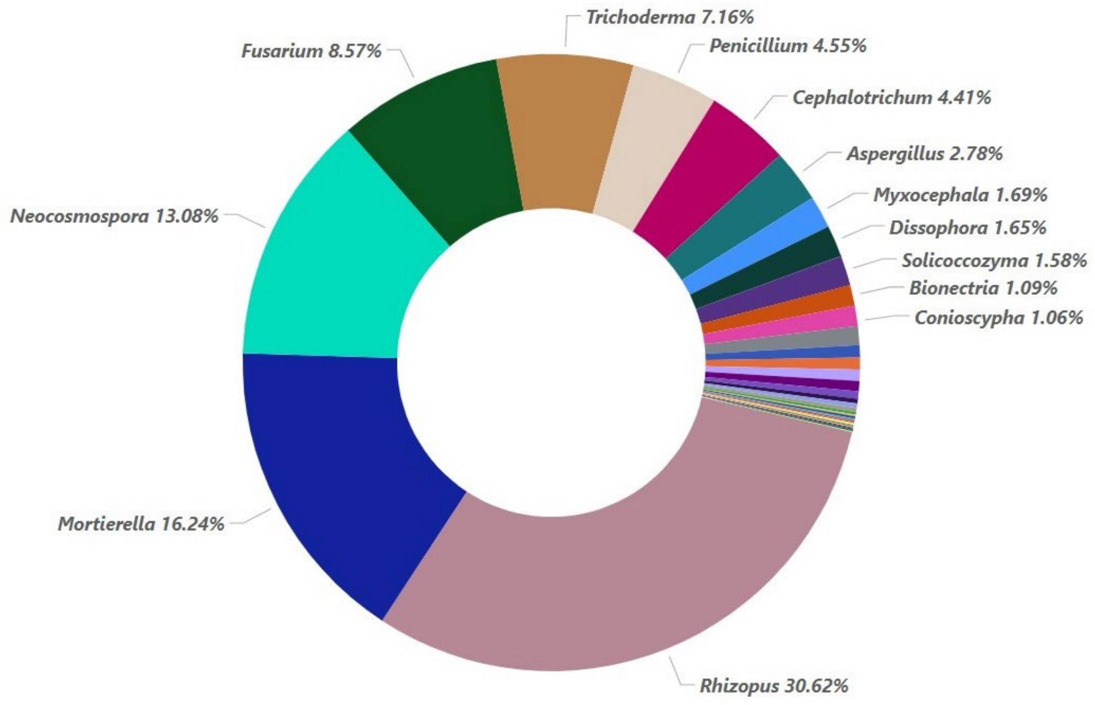

Figure S2. Relative abundance with percentages above 1% for Genera taxa, for sample M0.

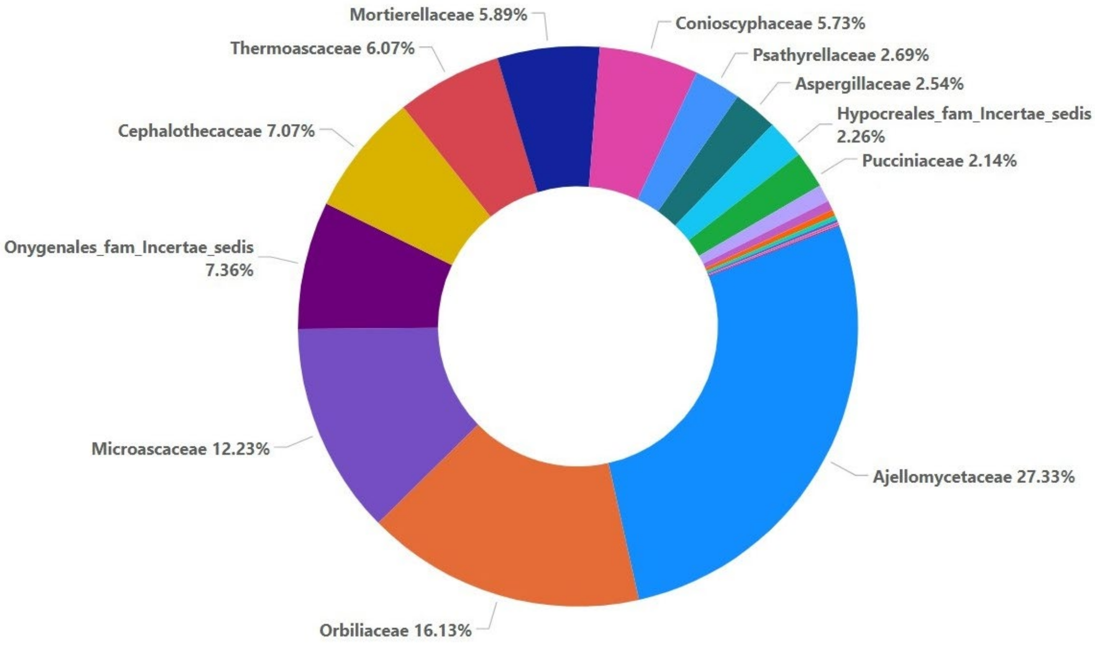

Figure S3. Relative abundance with percentages above 1% for Genera taxa, for sample A5.

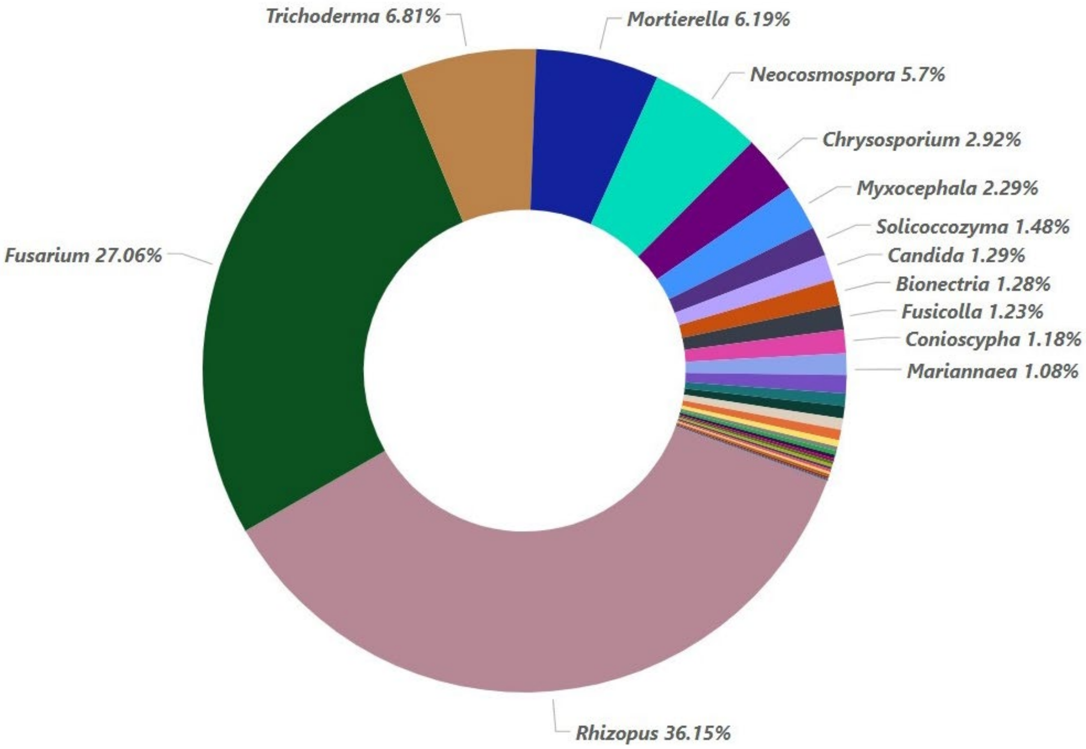

Figure S4. Relative abundance with percentages above 1% for Genera taxa, for sample M5.

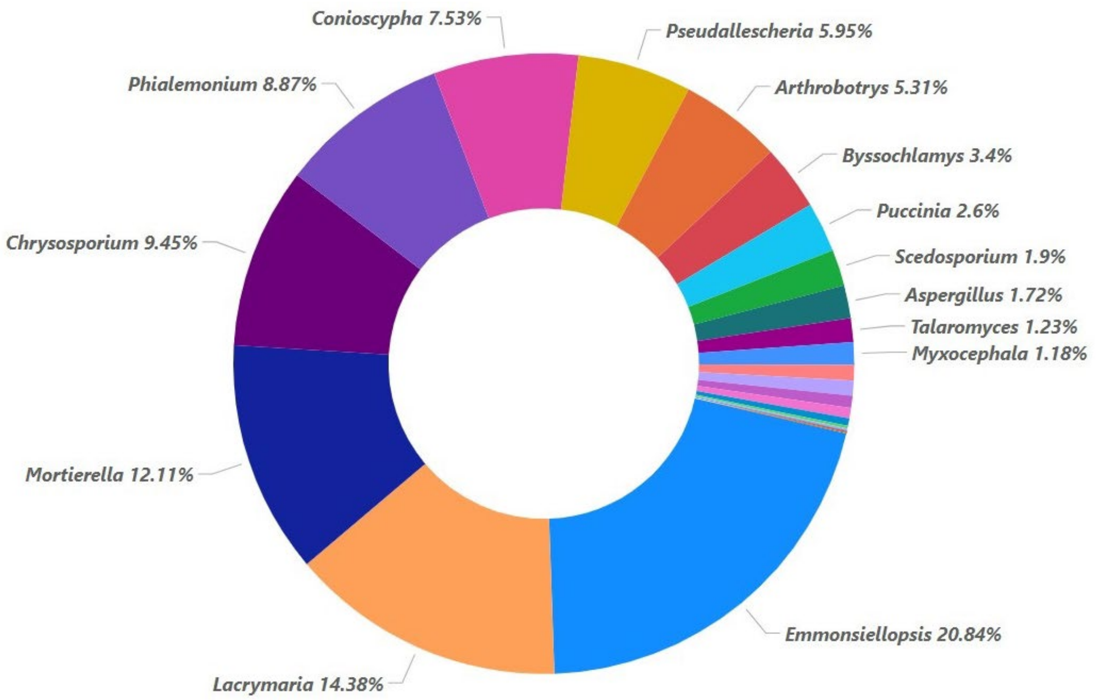

Figure S5. Relative abundance with percentages above 1% for Genera taxa, for sample A20.

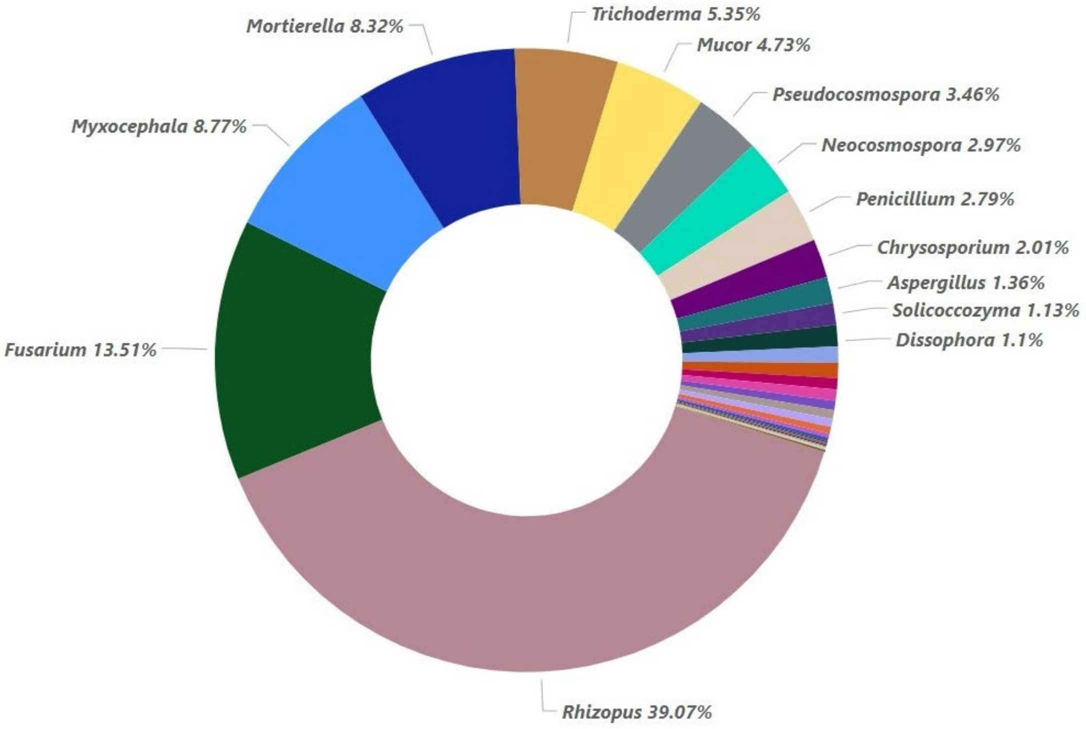

Figure S6. Relative abundance with percentages above 1% for Genera taxa, for sample M20.
